# Supplementary figures and images for: Robotic evaluation of a 3D-printed scaffold for reconstruction of scapholunate interosseous ligament rupture: a biomechanical cadaveric study
Source: PeerJ. 2025 Aug 20;13:e19766. doi: 10.7717/peerj.19766 (PMC12374688; doi:10.7717/peerj.19766)

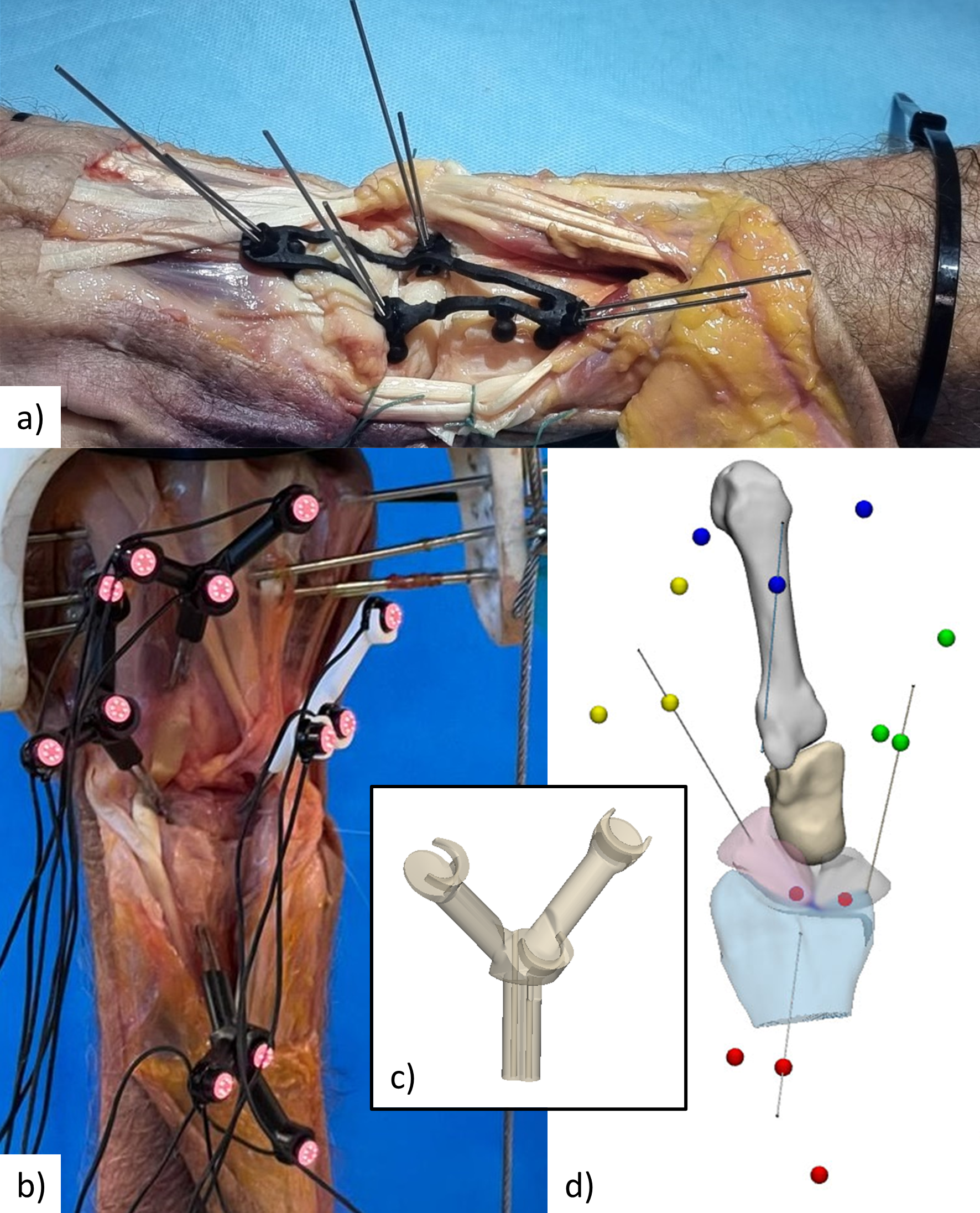

Supplement: Supplemental Information 1 — (A) Marker pins installed using placement guide. Placement guide was 3D-printed using stereolithography to ensure low error in pin hole formation. (B) Motion capture markers mounted on marker pins with wrist in neutral pose. (C) Motion capture marker holder. Transparency shows internal pin holes with one at set depth and the other free to pass through. A mirrored version was also used to fix any marker occlusion found during specimen manipulation. (D) Digital model in neutral pose with markers from a static trial also acquired in neutral pose. 3D –three-dimensional. [file peerj-13-19766-s001.png]

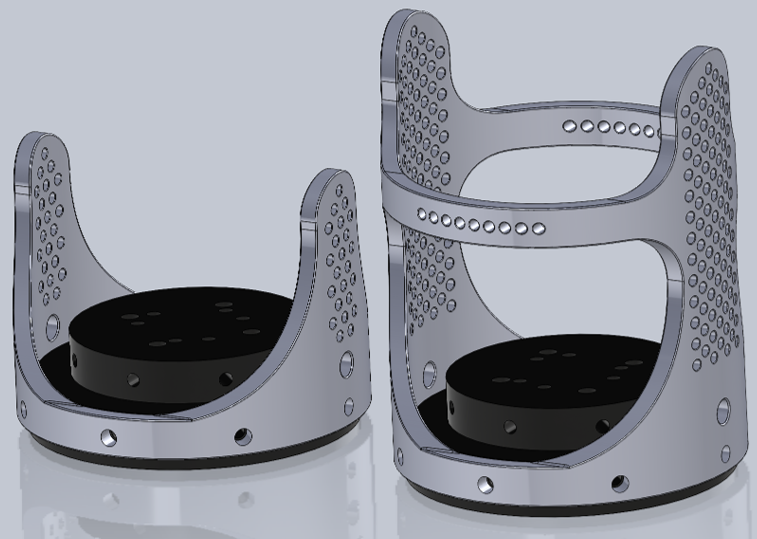

Supplement: Supplemental Information 2 — These custom mounting fixtures reduced the experiment time by one hour (i.e., no need to dissect down to bone, no potting of upper and lower portions of specimen, no transection at elbow), which was extremely helpful as the protocol was already ¿3 hours. Left: Upper mounting fixture. Right: Lower mounting fixture. [file peerj-13-19766-s002.png]

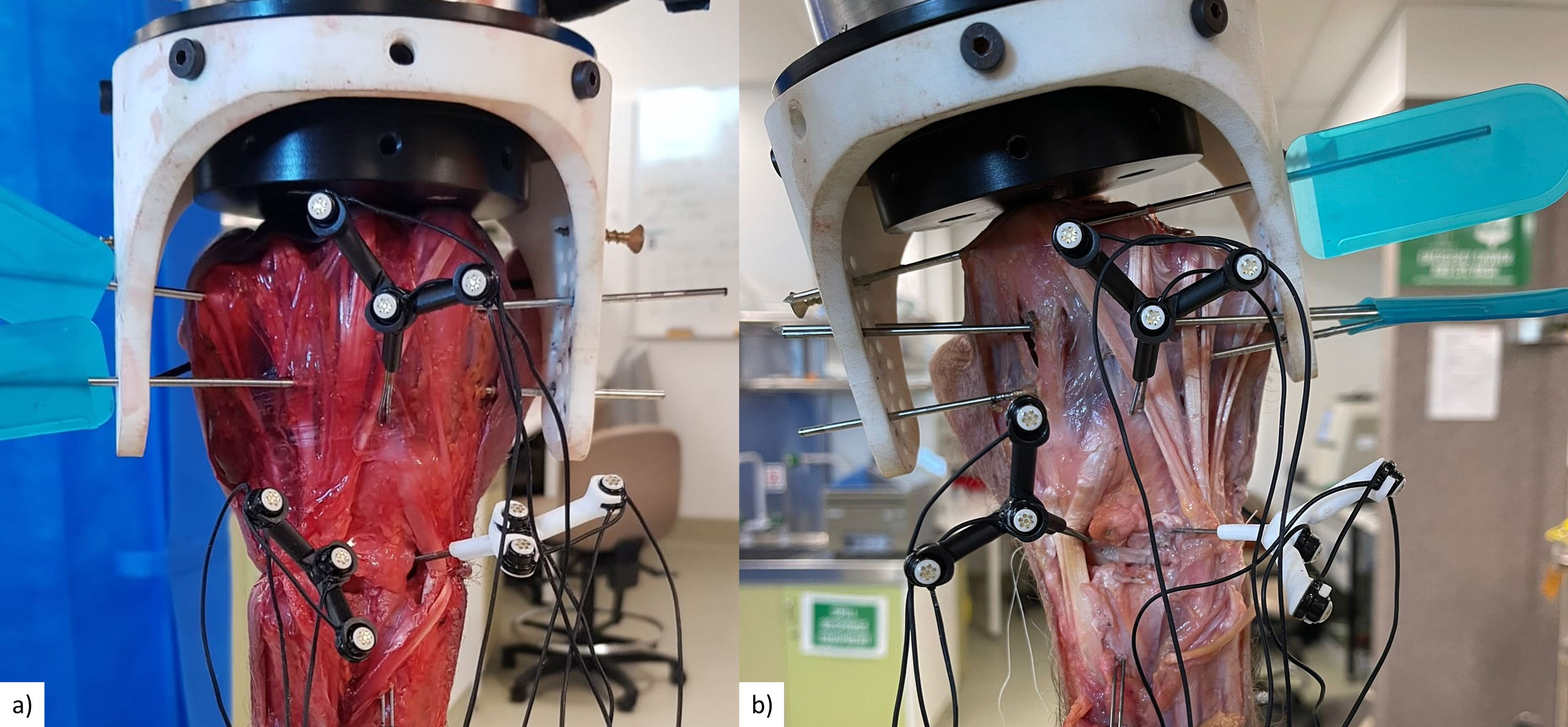

Supplement: Supplemental Information 3 — (A) A larger sized specimen fixed with two K-wires. (B) A smaller sized specimen fixed with four K-wires. [file peerj-13-19766-s003.png]

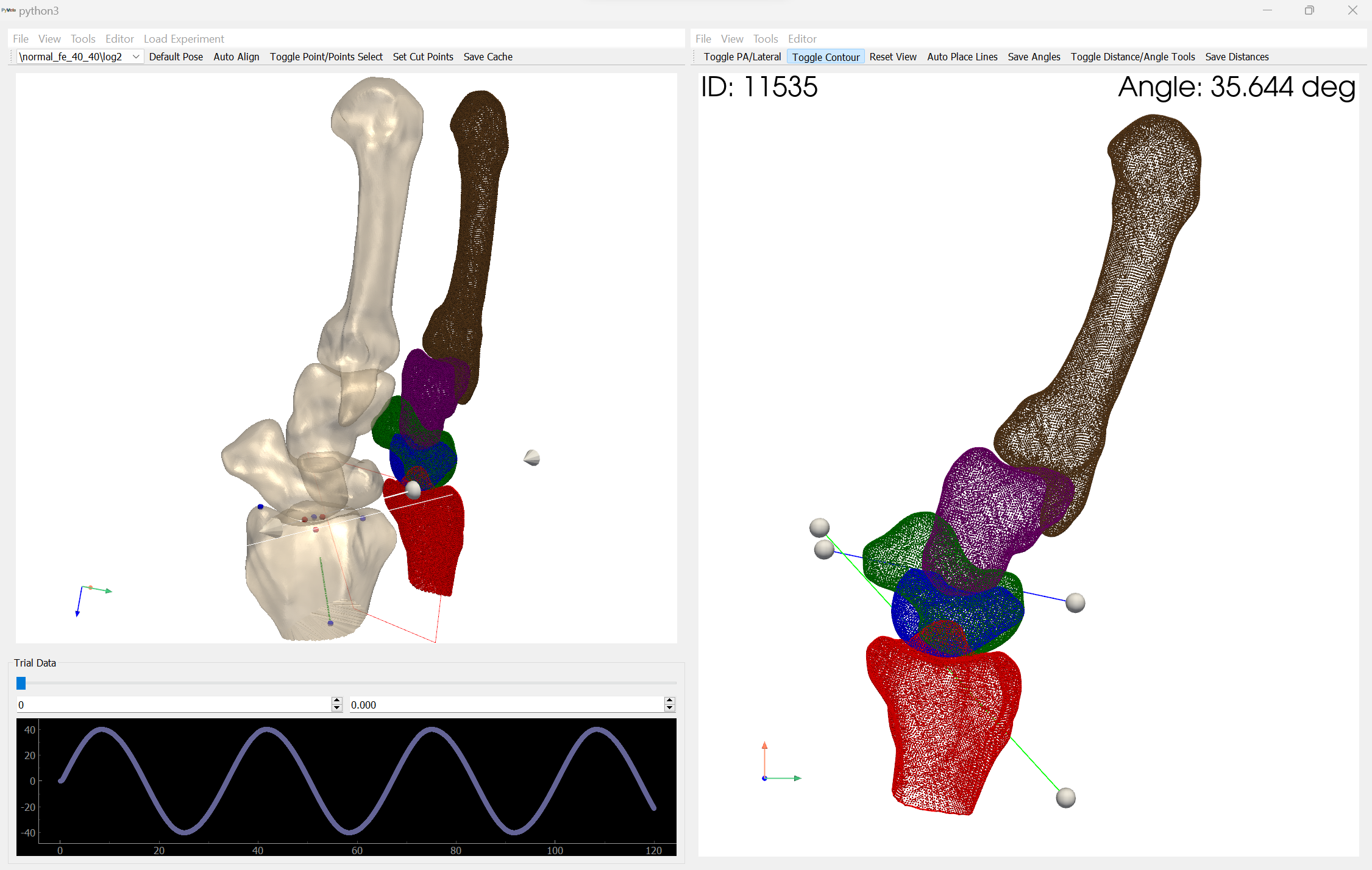

Supplement: Supplemental Information 4 — Left view: Manually placement of projection plane, or automatically find posterior/anterior plane or lateral plane based on methods from literature. Right view: Measure angles and distances in right hand view using interactive line tools. Method used (Badida, R., et al., An Approach to Robotic Testing of the Wrist Using Three-Dimensional Imaging and a Hybrid Testing Methodology. Journal of Biomechanical Engineering, 2020. 142(6).). Where it was subsequently used: (Badida, R., et al., The role of scapholunate interosseous, dorsal intercarpal, and radiolunate ligaments in wrist biomechanics. Journal of Biomechanics, 2021. 125: p. 110567.). Other methods found but were not used: (Shivdas, S., M. Hashim, and T.S. Ahmad, A three-dimensional virtual morphometry study of the sigmoid notch of the distal radius. Journal of Orthopaedic Surgery, 2018. 26(3): p. 2309499018802504.) and (Zhang, X., et al., Imaging study of coronal structural matching of the distal radius in normal adults. BMC Medical Imaging, 2020. 20(1): p. 35.). [file peerj-13-19766-s004.png]

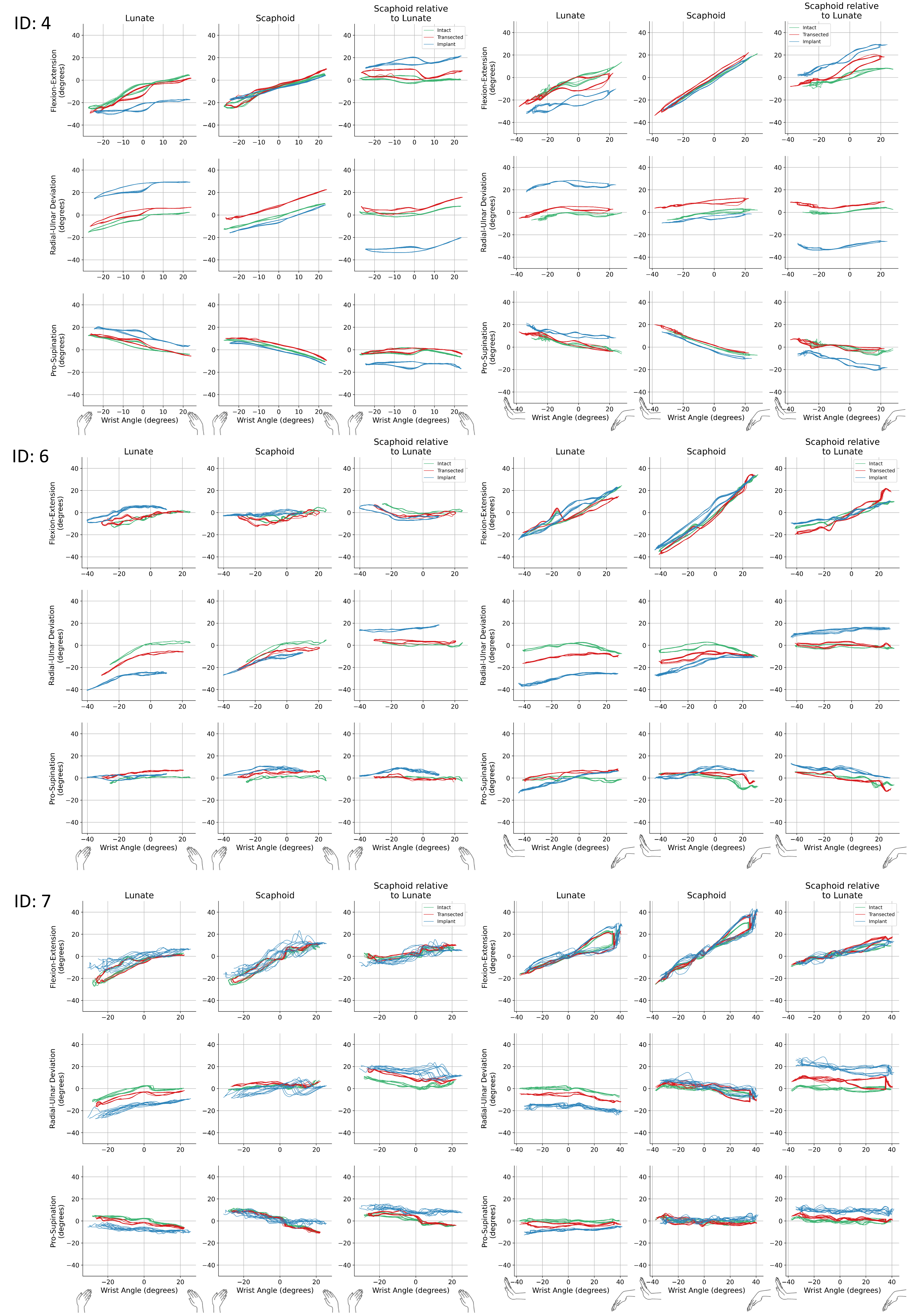

Supplement: Supplemental Information 5 — Left: radial-ulnar deviation, Right: flexion-extension. Green: intact, Red: transected, Blue: scaffold. ID –specimen identification number [file peerj-13-19766-s005.png]
