# Supplementary material for: Robotic evaluation of a 3D-printed scaffold for reconstruction of scapholunate interosseous ligament rupture: a biomechanical cadaveric study
Source: PeerJ. 2025 Aug 20;13:e19766. doi: 10.7717/peerj.19766 (PMC12374688; doi:10.7717/peerj.19766)
Supplement: Supplemental Information 6 — Discrete variables calculated form continuous time series data. Min: average of minimum values in each cycle. Max: average of maximum values in each cycle. Neutral: average of values at Neutral pose (i.e., first fame of each cycle). Values are relative to neutral position (i.e., first fame of intact) for each sample. [file peerj-13-19766-s006.docx]

| Sample | Configuration | Wrist External Angles | | | | | |
| --- | --- | --- | --- | --- | --- | --- | --- |
|  |  | Min | SD | Neutral | SD | Max | SD |
| 1 | Intact | Invalid Motion Capture | | | | | |
|  | Transected | Invalid Motion Capture | | | | | |
|  | Scaffold | Invalid Motion Capture | | | | | |
| 2 | Intact | -27.3 | 1.2 | 0.1 | 0.0 | 22.2 | 2.5 |
|  | Transected | -40.1 | 1.9 | -0.3 | 0.9 | 2.1 | 2.9 |
|  | Scaffold | Invalid Motion Capture | | | | | |
| 3 | Intact | -20.4 | 0.3 | 0.1 | 0.0 | 18.0 | 0.1 |
|  | Transected | Invalid Motion Capture | | | | | |
|  | Scaffold | Invalid Motion Capture | | | | | |
| 4 | Intact | -27.1 | 0.2 | 0.1 | 0.0 | 23.1 | 0.1 |
|  | Transected | -26.4 | 0.1 | 0.1 | 0.0 | 23.9 | 0.1 |
|  | Scaffold | -24.3 | 0.0 | 0.1 | 0.0 | 23.3 | 0.1 |
| 5 | Intact | -30.1 | 0.2 | 0.1 | 0.0 | 16.7 | 0.2 |
|  | Transected | -28.9 | 0.0 | 0.1 | 0.0 | 15.7 | 1.2 |
|  | Scaffold | Invalid Motion Capture | | | | | |
| 6 | Intact | -25.6 | 0.1 | 0.1 | 0.0 | 24.9 | 0.1 |
|  | Transected | -31.0 | 0.1 | 0.1 | 0.0 | 20.5 | 0.3 |
|  | Scaffold | -40.1 | 0.0 | 0.1 | 0.0 | 10.0 | 0.2 |
| 7 | Intact | -27.7 | 0.7 | 0.1 | 0.0 | 20.5 | 0.8 |
|  | Transected | -26.2 | 0.2 | 0.1 | 0.0 | 21.9 | 0.3 |
|  | Scaffold | -24.3 | 3.5 | 0.1 | 0.0 | 21.7 | 3.3 |
| 8 | Intact | -44.9 | 0.6 | 0.1 | 0.0 | 28.1 | 8.3 |
|  | Transected | -36.9 | 0.1 | 0.1 | 0.0 | 26.4 | 0.1 |
|  | Scaffold | Invalid Motion Capture | | | | | |
| 9 | Intact | -21.9 | 0.3 | 0.1 | 0.0 | 24.0 | 0.5 |
|  | Transected | -25.2 | 0.1 | 0.1 | 0.0 | 22.1 | 0.2 |
|  | Scaffold | Invalid Motion Capture | | | | | |
