# Supplementary material for: Robotic evaluation of a 3D-printed scaffold for reconstruction of scapholunate interosseous ligament rupture: a biomechanical cadaveric study
Source: PeerJ. 2025 Aug 20;13:e19766. doi: 10.7717/peerj.19766 (PMC12374688; doi:10.7717/peerj.19766)
Supplement: Supplemental Information 7 [file peerj-13-19766-s007.docx]

| Sample | Configuration | Flexion-Extension | | | | | | Radial-Ulnar Deviation | | | | | | Pro-Supination | | | | | |
| --- | --- | --- | --- | --- | --- | --- | --- | --- | --- | --- | --- | --- | --- | --- | --- | --- | --- | --- | --- |
|  |  | Min | SD | Neutral | SD | Max | SD | Min | SD | Neutral | SD | Max | SD | Min | SD | Neutral | SD | Max | SD |
| 1 | Intact | Invalid Motion Capture | | | | | | | | | | | | | | | | | |
|  | Transected | Invalid Motion Capture | | | | | | | | | | | | | | | | | |
|  | Scaffold | Invalid Motion Capture | | | | | | | | | | | | | | | | | |
| 2 | Intact | -17.5 | 0.9 | -1.1 | 1.3 | 30.9 | 1.1 | -9.5 | 0.1 | 2.3 | 0.5 | 11.7 | 1.2 | -11.0 | 0.7 | 1.8 | 0.4 | 5.0 | 0.6 |
|  | Transected | -20.6 | 1.5 | 25.2 | 2.7 | 30.2 | 1.2 | -9.8 | 1.2 | 4.3 | 3.0 | 10.3 | 1.3 | -14.8 | 0.9 | -12.1 | 2.4 | 6.2 | 4.3 |
|  | Scaffold | Invalid Motion Capture | | | | | | | | | | | | | | | | | |
| 3 | Intact | -18.3 | 0.8 | -0.4 | 0.8 | 11.5 | 0.4 | -15.4 | 0.1 | 0.8 | 0.8 | 4.4 | 0.4 | -14.7 | 0.2 | -0.3 | 0.2 | 14.2 | 0.3 |
|  | Transected | Invalid Motion Capture | | | | | | | | | | | | | | | | | |
|  | Scaffold | Invalid Motion Capture | | | | | | | | | | | | | | | | | |
| 4 | Intact | -24.2 | 0.8 | -5.7 | 0.7 | 5.2 | 0.4 | -12.8 | 0.2 | -1.4 | 0.8 | 10.0 | 0.2 | -10.5 | 0.2 | 1.8 | 0.3 | 8.9 | 0.4 |
|  | Transected | -23.3 | 0.9 | -4.7 | 0.9 | 10.0 | 0.1 | -4.0 | 0.2 | 7.8 | 0.6 | 22.5 | 0.1 | -9.4 | 0.1 | 4.7 | 0.3 | 10.5 | 0.2 |
|  | Scaffold | -17.3 | 0.3 | -7.1 | 0.2 | 4.1 | 0.1 | -15.9 | 0.1 | -5.7 | 1.1 | 8.9 | 0.1 | -13.2 | 0.1 | -0.8 | 0.2 | 6.7 | 0.7 |
| 5 | Intact | -31.0 | 0.5 | -0.5 | 0.8 | 5.0 | 0.5 | -5.1 | 0.5 | 0.8 | 0.8 | 8.5 | 0.2 | -10.1 | 0.5 | 0.4 | 2.6 | 14.0 | 0.3 |
|  | Transected | -29.7 | 0.3 | -1.0 | 2.6 | 16.3 | 4.1 | -3.6 | 0.1 | 0.8 | 0.2 | 8.6 | 0.8 | -13.6 | 1.2 | 0.1 | 1.4 | 12.4 | 0.2 |
|  | Scaffold | Invalid Motion Capture | | | | | | | | | | | | | | | | | |
| 6 | Intact | -8.0 | 1.3 | -4.5 | 0.7 | 3.6 | 1.2 | -15.1 | 0.1 | 2.0 | 0.3 | 4.7 | 0.1 | -3.7 | 0.5 | 1.5 | 0.3 | 2.2 | 0.1 |
|  | Transected | -10.8 | 1.7 | -5.6 | 1.7 | 1.3 | 1.3 | -22.6 | 0.2 | -4.3 | 0.9 | -2.1 | 0.4 | 0.5 | 0.3 | 5.3 | 0.6 | 7.0 | 0.5 |
|  | Scaffold | -3.4 | 0.4 | 0.6 | 1.6 | 1.0 | 1.4 | -27.0 | 0.1 | -10.0 | 1.1 | -6.6 | 0.2 | 2.4 | 0.1 | 8.3 | 1.0 | 9.9 | 0.9 |
| 7 | Intact | -23.9 | 2.0 | -2.3 | 0.4 | 10.3 | 0.6 | -1.3 | 0.3 | 3.4 | 0.4 | 6.9 | 1.0 | -10.1 | 0.2 | 3.2 | 0.4 | 10.6 | 0.8 |
|  | Transected | -22.3 | 1.5 | -2.8 | 0.4 | 11.6 | 0.7 | 1.5 | 0.5 | 4.7 | 0.8 | 6.5 | 0.2 | -11.0 | 0.2 | 2.6 | 0.5 | 9.2 | 0.4 |
|  | Scaffold | -15.4 | 3.1 | 4.1 | 3.9 | 14.6 | 3.7 | -7.4 | 1.1 | 1.6 | 1.3 | 5.4 | 3.1 | -3.8 | 1.5 | 0.1 | 1.5 | 9.6 | 1.2 |
| 8 | Intact | -9.6 | 1.6 | -0.2 | 0.8 | 16.1 | 1.4 | -23.8 | 0.5 | 0.2 | 0.2 | 8.6 | 7.9 | -14.2 | 2.1 | 0.8 | 0.3 | 5.1 | 0.4 |
|  | Transected | -23.9 | 0.4 | -15.0 | 0.8 | 4.1 | 0.2 | -22.5 | 0.2 | -3.2 | 0.3 | 4.3 | 0.8 | -30.8 | 0.3 | -14.1 | 0.6 | -11.4 | 1.0 |
|  | Scaffold | Invalid Motion Capture | | | | | | | | | | | | | | | | | |
| 9 | Intact | -18.3 | 0.5 | -1.0 | 1.8 | 10.4 | 0.9 | -1.8 | 0.7 | 3.6 | 0.9 | 16.3 | 2.2 | -2.6 | 0.5 | 1.8 | 1.0 | 5.1 | 1.1 |
|  | Transected | -15.6 | 0.3 | 4.8 | 1.6 | 14.2 | 0.4 | -3.4 | 1.1 | 4.5 | 2.5 | 23.3 | 6.1 | -1.7 | 0.5 | 5.3 | 1.4 | 6.7 | 0.2 |
|  | Scaffold | Invalid Motion Capture | | | | | | | | | | | | | | | | | |
