# Supplementary material for: Robotic evaluation of a 3D-printed scaffold for reconstruction of scapholunate interosseous ligament rupture: a biomechanical cadaveric study
Source: PeerJ. 2025 Aug 20;13:e19766. doi: 10.7717/peerj.19766 (PMC12374688; doi:10.7717/peerj.19766)
Supplement: Supplemental Information 8 [file peerj-13-19766-s008.docx]

| Sample | Configuration | Flexion-Extension | | | | | | Radial-Ulnar Deviation | | | | | | Pro-Supination | | | | | |
| --- | --- | --- | --- | --- | --- | --- | --- | --- | --- | --- | --- | --- | --- | --- | --- | --- | --- | --- | --- |
|  |  | Min | SD | Neutral | SD | Max | SD | Min | SD | Neutral | SD | Max | SD | Min | SD | Neutral | SD | Max | SD |
| 1 | Intact | Invalid Motion Capture | | | | | | | | | | | | | | | | | |
|  | Transected | Invalid Motion Capture | | | | | | | | | | | | | | | | | |
|  | Scaffold | Invalid Motion Capture | | | | | | | | | | | | | | | | | |
| 2 | Intact | -15.9 | 1.3 | -1.1 | 0.7 | 25.4 | 0.9 | -11.5 | 1.8 | 0.6 | 0.2 | 1.8 | 0.6 | -12.7 | 0.2 | -0.5 | 0.3 | 1.6 | 0.7 |
|  | Transected | -19.2 | 0.6 | 46.1 | 20.7 | 59.4 | 51.0 | -16.4 | 0.2 | -6.3 | 2.3 | -1.5 | 5.9 | -25.7 | 16.3 | -20.7 | 8.5 | 2.9 | 1.0 |
|  | Scaffold | Invalid Motion Capture | | | | | | | | | | | | | | | | | |
| 3 | Intact | -17.7 | 1.0 | 2.7 | 1.7 | 12.6 | 1.9 | 0.5 | 0.6 | 11.6 | 1.2 | 13.6 | 1.1 | -16.0 | 0.1 | 3.2 | 0.7 | 19.8 | 0.3 |
|  | Transected | Invalid Motion Capture | | | | | | | | | | | | | | | | | |
|  | Scaffold | Invalid Motion Capture | | | | | | | | | | | | | | | | | |
| 4 | Intact | -25.1 | 0.5 | -6.3 | 2.8 | 4.5 | 0.3 | -15.1 | 0.2 | -1.9 | 1.6 | 2.3 | 0.1 | -4.1 | 0.1 | 3.1 | 2.4 | 12.9 | 0.3 |
|  | Transected | -29.2 | 0.1 | -9.7 | 3.7 | 1.8 | 0.1 | -10.0 | 0.0 | 3.4 | 1.7 | 6.9 | 0.0 | -5.6 | 0.1 | 5.0 | 1.6 | 13.8 | 0.5 |
|  | Scaffold | -28.8 | 0.9 | -24.0 | 3.2 | -17.2 | 0.2 | 14.5 | 0.3 | 25.0 | 3.2 | 29.4 | 0.2 | 3.1 | 0.3 | 12.8 | 2.8 | 19.5 | 0.5 |
| 5 | Intact | -26.4 | 0.5 | -3.8 | 5.1 | 1.2 | 2.4 | -13.0 | 0.2 | -0.9 | 0.7 | 1.9 | 0.3 | -6.6 | 0.5 | 0.4 | 1.2 | 13.4 | 0.3 |
|  | Transected | -26.4 | 0.5 | -4.0 | 8.0 | 0.9 | 4.3 | -14.1 | 0.1 | -1.6 | 1.0 | -0.5 | 0.7 | -10.8 | 1.2 | 2.1 | 1.6 | 14.7 | 0.6 |
|  | Scaffold | Invalid Motion Capture | | | | | | | | | | | | | | | | | |
| 6 | Intact | -11.9 | 1.5 | -3.7 | 0.5 | 1.6 | 0.5 | -17.3 | 0.2 | 1.5 | 0.2 | 3.4 | 0.5 | -4.8 | 0.3 | 0.7 | 0.3 | 1.3 | 0.1 |
|  | Transected | -10.3 | 1.4 | -2.9 | 0.7 | 1.4 | 0.3 | -27.1 | 0.1 | -7.5 | 0.8 | -5.4 | 0.3 | -0.3 | 0.5 | 6.3 | 0.5 | 7.2 | 0.2 |
|  | Scaffold | -8.0 | 1.1 | 5.5 | 0.3 | 5.9 | 0.4 | -40.6 | 0.1 | -25.7 | 0.8 | -24.5 | 0.6 | -1.0 | 1.5 | 2.4 | 0.3 | 3.8 | 0.1 |
| 7 | Intact | -25.4 | 1.3 | -3.4 | 1.3 | 2.2 | 0.4 | -11.9 | 0.3 | 2.3 | 0.3 | 2.6 | 0.2 | -5.8 | 0.1 | 2.2 | 0.2 | 4.7 | 0.2 |
|  | Transected | -22.8 | 1.5 | -4.6 | 1.1 | 1.3 | 0.2 | -15.8 | 0.9 | -3.1 | 0.2 | -2.0 | 0.2 | -7.0 | 0.2 | -1.7 | 0.2 | 3.3 | 0.7 |
|  | Scaffold | -10.0 | 4.1 | 1.0 | 2.3 | 6.9 | 0.5 | -25.9 | 1.2 | -13.9 | 1.1 | -9.9 | 0.5 | -10.9 | 0.4 | -7.4 | 1.3 | -2.9 | 0.9 |
| 8 | Intact | -14.5 | 0.7 | 0.3 | 0.7 | 10.6 | 1.4 | -18.3 | 0.4 | 0.9 | 0.4 | 9.6 | 7.3 | -8.0 | 2.0 | 0.3 | 0.6 | 7.4 | 0.8 |
|  | Transected | -26.2 | 0.6 | -15.5 | 1.1 | 0.5 | 0.2 | -9.6 | 0.4 | 6.1 | 0.6 | 11.7 | 0.4 | -11.9 | 0.5 | 0.2 | 1.2 | 2.3 | 1.6 |
|  | Scaffold | Invalid Motion Capture | | | | | | | | | | | | | | | | | |
| 9 | Intact | -16.3 | 0.2 | -2.7 | 1.6 | 8.0 | 0.8 | -4.3 | 1.2 | 0.6 | 1.6 | 5.2 | 0.6 | -7.5 | 0.8 | -0.9 | 0.7 | 0.6 | 1.0 |
|  | Transected | -15.3 | 0.2 | -2.0 | 2.2 | 6.7 | 0.2 | -3.6 | 0.2 | 2.8 | 0.1 | 6.2 | 0.1 | -6.0 | 0.1 | -1.1 | 0.9 | -0.5 | 0.4 |
|  | Scaffold | Invalid Motion Capture | | | | | | | | | | | | | | | | | |
