# Supplementary material for: Robotic evaluation of a 3D-printed scaffold for reconstruction of scapholunate interosseous ligament rupture: a biomechanical cadaveric study
Source: PeerJ. 2025 Aug 20;13:e19766. doi: 10.7717/peerj.19766 (PMC12374688; doi:10.7717/peerj.19766)
Supplement: Supplemental Information 9 [file peerj-13-19766-s009.docx]

| Sample | Configuration | Flexion-Extension | | | | | | Radial-Ulnar Deviation | | | | | | Pro-Supination | | | | | |
| --- | --- | --- | --- | --- | --- | --- | --- | --- | --- | --- | --- | --- | --- | --- | --- | --- | --- | --- | --- |
|  |  | Min | SD | Neutral | SD | Max | SD | Min | SD | Neutral | SD | Max | SD | Min | SD | Neutral | SD | Max | SD |
| 1 | Intact | Invalid Motion Capture | | | | | | | | | | | | | | | | | |
|  | Transected | Invalid Motion Capture | | | | | | | | | | | | | | | | | |
|  | Scaffold | Invalid Motion Capture | | | | | | | | | | | | | | | | | |
| 2 | Intact | -3.2 | 1.3 | 0.0 | 0.7 | 7.5 | 2.0 | -0.6 | 0.8 | 1.7 | 0.5 | 13.3 | 1.1 | -1.1 | 1.3 | 2.3 | 0.2 | 4.2 | 0.3 |
|  | Transected | -34.3 | 51.3 | -20.9 | 19.8 | 8.7 | 2.3 | 1.2 | 4.6 | 10.6 | 3.1 | 16.8 | 1.4 | -5.9 | 0.5 | 8.6 | 9.1 | 14.3 | 17.2 |
|  | Scaffold | Invalid Motion Capture | | | | | | | | | | | | | | | | | |
| 3 | Intact | -4.1 | 0.7 | -3.1 | 0.9 | 1.0 | 0.2 | -16.4 | 0.3 | -10.8 | 0.4 | -9.1 | 1.5 | -8.5 | 0.6 | -3.5 | 0.8 | 1.9 | 0.7 |
|  | Transected | Invalid Motion Capture | | | | | | | | | | | | | | | | | |
|  | Scaffold | Invalid Motion Capture | | | | | | | | | | | | | | | | | |
| 4 | Intact | -1.9 | 1.0 | 0.5 | 3.1 | 2.7 | 1.3 | -0.6 | 1.1 | 0.5 | 0.9 | 7.8 | 0.1 | -6.5 | 0.2 | -1.3 | 2.6 | 0.8 | 1.2 |
|  | Transected | 1.0 | 0.6 | 5.0 | 4.5 | 9.1 | 0.8 | 2.3 | 1.7 | 4.4 | 1.1 | 15.7 | 0.1 | -3.9 | 0.2 | -0.3 | 1.8 | 1.2 | 0.3 |
|  | Scaffold | 10.7 | 0.2 | 16.9 | 3.3 | 21.5 | 0.1 | -32.0 | 1.5 | -30.7 | 2.1 | -20.3 | 0.1 | -17.0 | 0.2 | -13.6 | 2.9 | -11.3 | 0.8 |
| 5 | Intact | -5.7 | 0.8 | 3.3 | 5.1 | 8.7 | 0.8 | -0.1 | 1.1 | 1.6 | 0.6 | 10.3 | 0.4 | -4.7 | 1.2 | -0.1 | 3.5 | 2.4 | 1.2 |
|  | Transected | -5.4 | 1.8 | 2.9 | 5.4 | 19.6 | 3.4 | 0.8 | 0.9 | 2.4 | 1.1 | 12.9 | 0.4 | -8.2 | 2.1 | -2.0 | 3.0 | 2.1 | 1.6 |
|  | Scaffold | Invalid Motion Capture | | | | | | | | | | | | | | | | | |
| 6 | Intact | -1.2 | 0.3 | -0.9 | 0.2 | 8.2 | 0.1 | -1.5 | 0.1 | 0.4 | 0.1 | 2.6 | 0.2 | -2.8 | 0.1 | 0.8 | 0.1 | 1.5 | 0.2 |
|  | Transected | -4.2 | 0.5 | -2.8 | 1.2 | 6.5 | 0.4 | 1.3 | 0.4 | 3.2 | 0.6 | 4.9 | 0.5 | -2.1 | 0.5 | -1.0 | 0.5 | 2.3 | 1.2 |
|  | Scaffold | -6.2 | 0.9 | -4.8 | 1.5 | 5.6 | 1.5 | 12.7 | 0.7 | 15.7 | 0.3 | 18.4 | 0.1 | 1.7 | 0.2 | 5.9 | 0.8 | 9.2 | 0.2 |
| 7 | Intact | -2.9 | 0.6 | 1.1 | 1.1 | 8.6 | 0.4 | -0.2 | 0.7 | 1.1 | 0.6 | 10.8 | 0.5 | -4.9 | 0.2 | 1.1 | 0.4 | 6.5 | 1.2 |
|  | Transected | -3.4 | 1.5 | 1.8 | 1.4 | 10.5 | 0.5 | 5.3 | 1.1 | 7.8 | 0.7 | 18.1 | 0.5 | -4.1 | 0.2 | 4.4 | 0.4 | 8.1 | 1.3 |
|  | Scaffold | -7.6 | 1.0 | 3.1 | 3.8 | 8.6 | 3.4 | 9.3 | 1.8 | 15.5 | 2.2 | 20.1 | 2.0 | 5.6 | 0.8 | 7.5 | 0.7 | 13.8 | 1.4 |
| 8 | Intact | -0.8 | 0.2 | -0.5 | 0.2 | 6.3 | 0.3 | -5.5 | 0.2 | -0.8 | 0.4 | -0.2 | 0.2 | -7.3 | 0.1 | 0.5 | 0.4 | 1.3 | 0.3 |
|  | Transected | -1.4 | 0.3 | 0.5 | 0.4 | 4.0 | 0.3 | -13.1 | 0.2 | -9.3 | 0.5 | -5.9 | 1.9 | -18.9 | 0.3 | -14.3 | 1.5 | -11.4 | 2.0 |
|  | Scaffold | Invalid Motion Capture | | | | | | | | | | | | | | | | | |
| 9 | Intact | -3.2 | 0.6 | 1.6 | 0.3 | 5.4 | 0.5 | 0.5 | 1.5 | 3.0 | 1.3 | 12.7 | 3.4 | 0.3 | 1.5 | 2.7 | 0.5 | 6.7 | 0.6 |
|  | Transected | -0.6 | 0.5 | 6.8 | 0.6 | 10.3 | 0.8 | -1.0 | 1.4 | 1.6 | 2.5 | 18.1 | 6.6 | 0.7 | 0.6 | 6.4 | 0.5 | 8.7 | 1.9 |
|  | Scaffold | Invalid Motion Capture | | | | | | | | | | | | | | | | | |
