# Supplementary material for: Robotic evaluation of a 3D-printed scaffold for reconstruction of scapholunate interosseous ligament rupture: a biomechanical cadaveric study
Source: PeerJ. 2025 Aug 20;13:e19766. doi: 10.7717/peerj.19766 (PMC12374688; doi:10.7717/peerj.19766)
Supplement: Supplemental Information 10 — Values are relative to neutral position (i.e., first fame of intact) for each sample. [file peerj-13-19766-s010.docx]

| Sample | Configuration | Wrist External Angles | | | | | |
| --- | --- | --- | --- | --- | --- | --- | --- |
|  |  | Min | SD | Neutral | SD | Max | SD |
| 1 | Intact | Invalid Motion Capture | | | | | |
|  | Transected | Invalid Motion Capture | | | | | |
|  | Scaffold | Invalid Motion Capture | | | | | |
| 2 | Intact | -34.8 | 0.9 | 0.1 | 0.0 | 37.1 | 1.1 |
|  | Transected | -42.7 | 1.6 | 0.1 | 0.0 | 28.2 | 2.0 |
|  | Scaffold | Invalid Motion Capture | | | | | |
| 3 | Intact | -28.7 | 1.1 | 0.1 | 0.0 | 33.1 | 5.7 |
|  | Transected | Invalid Motion Capture | | | | | |
|  | Scaffold | Invalid Motion Capture | | | | | |
| 4 | Intact | -25.5 | 2.9 | 0.1 | 0.0 | 23.0 | 3.1 |
|  | Transected | -35.7 | 2.1 | 0.1 | 0.0 | 21.0 | 1.1 |
|  | Scaffold | -31.1 | 2.1 | 0.1 | 0.0 | 21.3 | 1.8 |
| 5 | Intact | -36.3 | 1.4 | 0.1 | 0.0 | 33.0 | 2.0 |
|  | Transected | -36.9 | 1.8 | 0.1 | 0.0 | 33.3 | 2.1 |
|  | Scaffold | Invalid Motion Capture | | | | | |
| 6 | Intact | -40.6 | 0.4 | 0.1 | 0.0 | 30.3 | 0.5 |
|  | Transected | -40.0 | 0.7 | 0.1 | 0.0 | 28.4 | 0.4 |
|  | Scaffold | -42.8 | 1.0 | 0.1 | 0.0 | 27.6 | 0.7 |
| 7 | Intact | -34.8 | 2.5 | 0.1 | 0.0 | 36.9 | 2.5 |
|  | Transected | -34.0 | 2.6 | 0.1 | 0.0 | 37.8 | 2.5 |
|  | Scaffold | -31.5 | 1.9 | 0.1 | 0.0 | 39.0 | 1.7 |
| 8 | Intact | -38.4 | 1.4 | 0.1 | 0.0 | 22.3 | 0.5 |
|  | Transected | -44.6 | 1.0 | 0.1 | 0.0 | 32.1 | 2.4 |
|  | Scaffold | Invalid Motion Capture | | | | | |
| 9 | Intact | -35.3 | 2.2 | 0.1 | 0.0 | 36.8 | 3.3 |
|  | Transected | -34.0 | 2.8 | 0.1 | 0.0 | 39.6 | 3.4 |
|  | Scaffold | Invalid Motion Capture | | | | | |
