# Supplementary material for: Robotic evaluation of a 3D-printed scaffold for reconstruction of scapholunate interosseous ligament rupture: a biomechanical cadaveric study
Source: PeerJ. 2025 Aug 20;13:e19766. doi: 10.7717/peerj.19766 (PMC12374688; doi:10.7717/peerj.19766)
Supplement: Supplemental Information 11 [file peerj-13-19766-s011.docx]

| Sample | Configuration | Flexion-Extension | | | | | | Radial-Ulnar Deviation | | | | | | Pro-Supination | | | | | |
| --- | --- | --- | --- | --- | --- | --- | --- | --- | --- | --- | --- | --- | --- | --- | --- | --- | --- | --- | --- |
|  |  | Min | SD | Neutral | SD | Max | SD | Min | SD | Neutral | SD | Max | SD | Min | SD | Neutral | SD | Max | SD |
| 1 | Intact | Invalid Motion Capture | | | | | | | | | | | | | | | | | |
|  | Transected | Invalid Motion Capture | | | | | | | | | | | | | | | | | |
|  | Scaffold | Invalid Motion Capture | | | | | | | | | | | | | | | | | |
| 2 | Intact | -15.9 | 1.2 | -0.9 | 0.5 | 19.2 | 1.6 | -1.5 | 0.4 | 0.5 | 0.2 | 1.2 | 0.2 | -4.7 | 1.3 | 0.8 | 0.2 | 5.2 | 0.3 |
|  | Transected | -18.3 | 1.3 | 2.3 | 0.9 | 17.5 | 2.0 | -0.4 | 0.2 | 2.2 | 0.2 | 5.4 | 0.3 | -7.6 | 2.0 | -3.5 | 0.3 | 5.0 | 0.5 |
|  | Scaffold | Invalid Motion Capture | | | | | | | | | | | | | | | | | |
| 3 | Intact | -21.0 | 0.9 | 0.7 | 0.9 | 20.8 | 0.6 | -19.7 | 1.5 | -1.0 | 1.4 | 8.7 | 0.5 | -4.5 | 0.3 | -0.6 | 0.6 | 11.9 | 1.2 |
|  | Transected | Invalid Motion Capture | | | | | | | | | | | | | | | | | |
|  | Scaffold | Invalid Motion Capture | | | | | | | | | | | | | | | | | |
| 4 | Intact | -22.5 | 2.9 | -0.9 | 1.1 | 17.1 | 2.7 | -6.0 | 0.7 | -0.2 | 1.2 | 2.1 | 1.0 | -7.2 | 0.2 | -0.3 | 0.2 | 12.9 | 1.2 |
|  | Transected | -30.4 | 2.7 | 1.9 | 1.3 | 20.5 | 1.5 | 4.0 | 0.3 | 9.2 | 1.8 | 11.9 | 0.8 | -5.2 | 0.1 | 1.6 | 0.2 | 19.5 | 0.4 |
|  | Scaffold | -27.1 | 2.0 | -2.2 | 1.6 | 16.2 | 2.0 | -9.3 | 0.2 | -4.6 | 1.7 | -1.8 | 0.5 | -10.6 | 0.1 | -3.7 | 0.3 | 12.9 | 0.4 |
| 5 | Intact | -30.9 | 2.2 | 2.7 | 0.9 | 16.9 | 1.7 | -5.4 | 0.4 | -0.9 | 0.3 | 11.0 | 0.7 | -3.5 | 0.3 | 1.4 | 0.4 | 7.2 | 1.7 |
|  | Transected | -33.2 | 2.5 | 1.9 | 0.5 | 15.6 | 1.9 | -6.9 | 0.5 | -2.2 | 0.5 | 10.8 | 0.5 | -7.4 | 0.3 | -1.4 | 0.5 | 6.8 | 0.9 |
|  | Scaffold | Invalid Motion Capture | | | | | | | | | | | | | | | | | |
| 6 | Intact | -34.1 | 1.0 | -1.6 | 1.0 | 33.1 | 0.7 | -9.9 | 0.3 | 1.1 | 0.6 | 1.7 | 1.2 | -10.0 | 0.5 | 0.6 | 0.9 | 4.3 | 0.3 |
|  | Transected | -36.1 | 1.3 | -3.6 | 1.0 | 34.2 | 0.2 | -16.0 | 0.4 | -7.0 | 1.3 | -5.9 | 0.7 | -4.8 | 0.4 | 3.6 | 1.2 | 5.3 | 0.3 |
|  | Scaffold | -32.0 | 1.3 | 7.2 | 3.4 | 32.4 | 1.0 | -27.1 | 0.5 | -12.1 | 0.4 | -9.5 | 0.9 | -0.1 | 0.7 | 9.0 | 1.5 | 9.5 | 1.4 |
| 7 | Intact | -22.8 | 2.4 | 0.9 | 0.7 | 27.7 | 5.9 | -5.9 | 1.7 | 0.1 | 1.1 | 3.1 | 0.5 | -3.4 | 0.6 | -0.8 | 1.3 | 4.1 | 1.3 |
|  | Transected | -21.6 | 2.7 | 2.0 | 1.2 | 32.2 | 7.3 | -6.8 | 5.0 | 2.0 | 0.7 | 5.9 | 0.8 | -3.8 | 1.0 | -1.8 | 2.0 | 4.6 | 1.7 |
|  | Scaffold | -17.4 | 3.0 | 3.8 | 2.2 | 32.6 | 9.3 | -4.6 | 4.0 | 1.4 | 1.6 | 8.4 | 2.0 | -2.4 | 0.7 | -0.1 | 1.1 | 4.9 | 1.3 |
| 8 | Intact | -27.8 | 1.4 | 2.7 | 0.7 | 24.7 | 0.5 | -8.4 | 0.2 | -0.5 | 0.4 | 0.0 | 0.4 | -12.5 | 0.1 | -1.8 | 0.9 | 17.6 | 0.3 |
|  | Transected | -38.7 | 2.4 | -6.2 | 0.4 | 12.9 | 1.7 | -4.9 | 0.3 | 2.4 | 1.6 | 3.0 | 1.2 | -28.1 | 1.4 | -18.6 | 0.4 | 7.4 | 0.8 |
|  | Scaffold | Invalid Motion Capture | | | | | | | | | | | | | | | | | |
| 9 | Intact | -28.2 | 2.8 | 0.9 | 1.2 | 27.6 | 2.1 | -1.6 | 1.3 | 1.6 | 2.7 | 4.2 | 1.8 | -1.1 | 1.8 | 1.2 | 0.8 | 5.1 | 1.7 |
|  | Transected | -27.1 | 2.9 | 3.7 | 1.3 | 39.5 | 4.0 | -2.2 | 2.9 | 5.4 | 1.7 | 8.0 | 0.5 | 0.6 | 0.4 | 4.0 | 0.8 | 6.6 | 0.7 |
|  | Scaffold | Invalid Motion Capture | | | | | | | | | | | | | | | | | |
