# Supplementary material for: Robotic evaluation of a 3D-printed scaffold for reconstruction of scapholunate interosseous ligament rupture: a biomechanical cadaveric study
Source: PeerJ. 2025 Aug 20;13:e19766. doi: 10.7717/peerj.19766 (PMC12374688; doi:10.7717/peerj.19766)
Supplement: Supplemental Information 12 [file peerj-13-19766-s012.docx]

| Sample | Configuration | Flexion-Extension | | | | | | Radial-Ulnar Deviation | | | | | | Pro-Supination | | | | | |
| --- | --- | --- | --- | --- | --- | --- | --- | --- | --- | --- | --- | --- | --- | --- | --- | --- | --- | --- | --- |
|  |  | Min | SD | Neutral | SD | Max | SD | Min | SD | Neutral | SD | Max | SD | Min | SD | Neutral | SD | Max | SD |
| 1 | Intact | Invalid Motion Capture | | | | | | | | | | | | | | | | | |
|  | Transected | Invalid Motion Capture | | | | | | | | | | | | | | | | | |
|  | Scaffold | Invalid Motion Capture | | | | | | | | | | | | | | | | | |
| 2 | Intact | -12.2 | 2.8 | -3.7 | 3.6 | 16.6 | 2.4 | -0.9 | 2.9 | 3.1 | 2.6 | 4.2 | 2.9 | -7.0 | 1.6 | 0.7 | 1.1 | 4.2 | 1.4 |
|  | Transected | -10.7 | 3.8 | -1.1 | 0.2 | 15.0 | 1.2 | -10.0 | 0.9 | -5.5 | 0.8 | -3.1 | 1.2 | -3.5 | 1.3 | -0.2 | 0.3 | 2.1 | 1.2 |
|  | Scaffold | Invalid Motion Capture | | | | | | | | | | | | | | | | | |
| 3 | Intact | -17.9 | 2.5 | 1.8 | 3.0 | 16.6 | 1.7 | -7.4 | 5.1 | 5.1 | 5.3 | 10.6 | 5.4 | -1.6 | 1.7 | 0.8 | 1.8 | 6.8 | 1.2 |
|  | Transected | Invalid Motion Capture | | | | | | | | | | | | | | | | | |
|  | Scaffold | Invalid Motion Capture | | | | | | | | | | | | | | | | | |
| 4 | Intact | -15.5 | 2.7 | -2.4 | 3.7 | 9.1 | 2.9 | -6.0 | 1.1 | -1.1 | 1.1 | -0.3 | 0.3 | -3.0 | 1.7 | 1.1 | 1.6 | 9.7 | 1.5 |
|  | Transected | -23.0 | 2.1 | -5.7 | 6.1 | 1.8 | 1.9 | -4.9 | 0.2 | 3.4 | 1.7 | 4.9 | 0.4 | -3.7 | 0.2 | 2.7 | 2.2 | 13.0 | 0.8 |
|  | Scaffold | -29.3 | 2.1 | -20.4 | 4.8 | -12.9 | 2.1 | 20.5 | 1.5 | 25.6 | 2.4 | 28.0 | 0.3 | 8.5 | 1.0 | 10.4 | 2.2 | 18.2 | 1.6 |
| 5 | Intact | -14.6 | 1.2 | 3.0 | 0.6 | 7.6 | 2.1 | -7.2 | 1.0 | -1.8 | 0.2 | 2.5 | 0.3 | -1.9 | 0.6 | -1.1 | 0.9 | 4.3 | 0.7 |
|  | Transected | -14.4 | 1.7 | 3.7 | 0.7 | 7.0 | 2.5 | -8.2 | 1.0 | -3.2 | 0.1 | 1.1 | 0.2 | -3.2 | 0.3 | -1.8 | 0.8 | 4.2 | 0.1 |
|  | Scaffold | Invalid Motion Capture | | | | | | | | | | | | | | | | | |
| 6 | Intact | -20.9 | 0.2 | -1.2 | 0.8 | 23.4 | 0.6 | -7.4 | 0.2 | 1.9 | 0.7 | 2.3 | 0.4 | -3.5 | 0.8 | 0.5 | 0.6 | 1.4 | 0.3 |
|  | Transected | -17.6 | 0.4 | -1.3 | 1.4 | 14.1 | 0.5 | -16.2 | 0.2 | -8.5 | 0.4 | -6.8 | 0.3 | -2.2 | 0.3 | 5.7 | 1.1 | 8.1 | 0.2 |
|  | Scaffold | -22.3 | 1.6 | 8.5 | 2.5 | 22.4 | 0.8 | -36.8 | 0.3 | -26.4 | 0.9 | -25.0 | 0.4 | -11.9 | 1.1 | 2.0 | 0.3 | 6.6 | 0.1 |
| 7 | Intact | -15.1 | 1.1 | -0.7 | 0.8 | 17.4 | 6.9 | -6.4 | 0.7 | 0.0 | 1.3 | 1.7 | 0.6 | -1.4 | 0.5 | -0.2 | 1.0 | 1.9 | 0.2 |
|  | Transected | -15.2 | 1.3 | -0.5 | 0.9 | 14.8 | 7.5 | -12.5 | 0.2 | -5.4 | 1.6 | -4.0 | 0.7 | -5.2 | 1.2 | -3.3 | 1.1 | -0.9 | 0.5 |
|  | Scaffold | -11.7 | 2.6 | 1.0 | 2.2 | 19.1 | 10.1 | -21.4 | 1.3 | -16.3 | 0.9 | -13.6 | 0.6 | -11.4 | 0.6 | -8.2 | 0.4 | -4.8 | 0.3 |
| 8 | Intact | -17.5 | 1.5 | 1.4 | 1.1 | 17.8 | 0.8 | -5.5 | 0.5 | 1.2 | 0.4 | 1.6 | 0.4 | -6.7 | 0.1 | -1.1 | 0.5 | 5.1 | 0.5 |
|  | Transected | -24.1 | 3.5 | -6.7 | 1.2 | 10.6 | 1.9 | 2.3 | 1.1 | 8.8 | 1.3 | 11.2 | 0.3 | -12.1 | 1.6 | -4.7 | 0.4 | 2.8 | 1.0 |
|  | Scaffold | Invalid Motion Capture | | | | | | | | | | | | | | | | | |
| 9 | Intact | -14.7 | 1.8 | -0.6 | 1.3 | 17.3 | 1.6 | 1.0 | 0.9 | 1.4 | 1.0 | 3.9 | 0.7 | -5.0 | 0.8 | 0.2 | 1.0 | 2.4 | 0.7 |
|  | Transected | -15.3 | 1.6 | -3.1 | 1.6 | 10.1 | 1.7 | 0.6 | 0.3 | 2.8 | 0.2 | 4.7 | 0.4 | -5.0 | 0.7 | -1.8 | 1.0 | 2.5 | 3.5 |
|  | Scaffold | Invalid Motion Capture | | | | | | | | | | | | | | | | | |
