# Supplementary material for: Robotic evaluation of a 3D-printed scaffold for reconstruction of scapholunate interosseous ligament rupture: a biomechanical cadaveric study
Source: PeerJ. 2025 Aug 20;13:e19766. doi: 10.7717/peerj.19766 (PMC12374688; doi:10.7717/peerj.19766)
Supplement: Supplemental Information 13 [file peerj-13-19766-s013.docx]

| Sample | Configuration | Flexion-Extension | | | | | | Radial-Ulnar Deviation | | | | | | Pro-Supination | | | | | |
| --- | --- | --- | --- | --- | --- | --- | --- | --- | --- | --- | --- | --- | --- | --- | --- | --- | --- | --- | --- |
|  |  | Min | SD | Neutral | SD | Max | SD | Min | SD | Neutral | SD | Max | SD | Min | SD | Neutral | SD | Max | SD |
| 1 | Intact | Invalid Motion Capture | | | | | | | | | | | | | | | | | |
|  | Transected | Invalid Motion Capture | | | | | | | | | | | | | | | | | |
|  | Scaffold | Invalid Motion Capture | | | | | | | | | | | | | | | | | |
| 2 | Intact | -5.6 | 3.0 | 2.8 | 3.8 | 8.6 | 3.5 | -4.1 | 3.0 | -2.6 | 2.7 | 0.4 | 2.9 | -1.8 | 1.0 | 0.1 | 1.2 | 3.9 | 0.7 |
|  | Transected | -11.9 | 2.0 | 3.4 | 1.0 | 7.7 | 0.6 | 3.0 | 1.7 | 7.8 | 0.7 | 14.4 | 0.2 | -6.5 | 1.1 | -3.3 | 0.4 | 5.4 | 1.4 |
|  | Scaffold | Invalid Motion Capture | | | | | | | | | | | | | | | | | |
| 3 | Intact | -7.1 | 0.7 | -1.1 | 2.2 | 4.7 | 1.2 | -13.1 | 5.5 | -6.1 | 6.2 | 2.9 | 2.7 | -6.2 | 1.6 | -1.5 | 2.2 | 6.6 | 1.1 |
|  | Transected | Invalid Motion Capture | | | | | | | | | | | | | | | | | |
|  | Scaffold | Invalid Motion Capture | | | | | | | | | | | | | | | | | |
| 4 | Intact | -7.5 | 1.1 | 1.6 | 2.7 | 8.3 | 0.1 | -1.3 | 0.4 | 0.8 | 0.2 | 4.0 | 0.3 | -6.3 | 0.9 | -1.4 | 1.7 | 5.2 | 1.5 |
|  | Transected | -7.5 | 0.4 | 7.6 | 4.8 | 19.3 | 0.4 | 3.4 | 0.1 | 5.9 | 0.2 | 9.3 | 0.2 | -2.6 | 1.1 | -1.1 | 2.1 | 7.4 | 0.3 |
|  | Scaffold | 1.8 | 0.5 | 18.2 | 3.2 | 29.5 | 0.1 | -33.4 | 0.3 | -30.3 | 0.8 | -25.5 | 0.1 | -20.5 | 0.2 | -14.1 | 2.5 | -3.8 | 0.7 |
| 5 | Intact | -16.4 | 1.2 | -0.3 | 0.8 | 10.0 | 0.4 | -0.5 | 0.5 | 0.9 | 0.5 | 8.7 | 0.7 | -6.9 | 0.2 | 2.5 | 1.0 | 4.8 | 0.4 |
|  | Transected | -19.2 | 1.2 | -1.8 | 0.5 | 9.4 | 0.3 | -0.9 | 0.8 | 1.0 | 0.5 | 10.0 | 0.5 | -11.3 | 0.3 | 0.3 | 1.2 | 5.3 | 0.3 |
|  | Scaffold | Invalid Motion Capture | | | | | | | | | | | | | | | | | |
| 6 | Intact | -13.3 | 0.9 | -0.4 | 1.8 | 9.9 | 0.3 | -2.9 | 0.5 | -0.8 | 1.3 | 0.0 | 1.1 | -7.7 | 1.1 | 0.2 | 0.4 | 5.7 | 0.1 |
|  | Transected | -18.5 | 1.0 | -2.3 | 2.4 | 21.4 | 0.2 | -2.0 | 0.7 | 1.6 | 1.6 | 2.3 | 1.2 | -11.8 | 0.2 | -2.1 | 0.3 | 5.6 | 0.3 |
|  | Scaffold | -10.2 | 0.3 | -1.3 | 1.0 | 10.2 | 0.3 | 8.7 | 1.3 | 14.3 | 1.3 | 16.0 | 0.4 | -0.1 | 0.1 | 7.0 | 1.7 | 12.3 | 0.6 |
| 7 | Intact | -7.8 | 1.5 | 1.6 | 1.4 | 11.2 | 0.2 | -2.5 | 0.7 | 0.1 | 0.9 | 2.7 | 0.5 | -3.0 | 0.4 | -0.6 | 0.7 | 3.6 | 0.5 |
|  | Transected | -6.4 | 1.4 | 2.5 | 0.6 | 17.6 | 0.4 | 2.7 | 3.5 | 7.4 | 1.1 | 11.1 | 0.5 | -1.0 | 0.4 | 1.5 | 0.9 | 6.8 | 1.1 |
|  | Scaffold | -6.7 | 0.6 | 2.8 | 1.6 | 14.3 | 0.4 | 14.9 | 3.1 | 17.7 | 1.1 | 24.0 | 2.6 | 5.5 | 0.8 | 8.1 | 1.2 | 12.7 | 1.1 |
| 8 | Intact | -10.9 | 0.3 | 1.3 | 0.4 | 7.1 | 0.2 | -3.9 | 0.5 | -1.6 | 0.5 | 0.5 | 0.3 | -5.9 | 0.2 | -0.6 | 0.6 | 12.7 | 0.2 |
|  | Transected | -16.2 | 0.5 | 0.5 | 1.6 | 3.0 | 0.5 | -11.1 | 0.3 | -6.4 | 2.8 | -3.2 | 0.8 | -16.3 | 0.4 | -13.9 | 0.3 | 4.8 | 0.3 |
|  | Scaffold | Invalid Motion Capture | | | | | | | | | | | | | | | | | |
| 9 | Intact | -13.5 | 1.8 | 1.5 | 1.7 | 10.4 | 1.8 | -4.2 | 1.3 | 0.2 | 2.7 | 1.6 | 2.1 | 0.1 | 1.2 | 1.0 | 0.9 | 6.0 | 1.1 |
|  | Transected | -11.8 | 1.3 | 6.7 | 0.5 | 29.5 | 2.2 | -5.7 | 2.7 | 2.5 | 1.9 | 5.7 | 0.8 | 2.4 | 2.1 | 5.7 | 0.3 | 9.4 | 0.3 |
|  | Scaffold | Invalid Motion Capture | | | | | | | | | | | | | | | | | |
